# The impact of negative and positive affectivity on the relationship between work-related psychological factors and work engagement in Japanese workers: a comparison of psychological distress

**DOI:** 10.1186/s40359-023-01250-y

**Published:** 2023-08-03

**Authors:** Toshiki Fukuzaki, Noboru Iwata

**Affiliations:** 1 Department of Clinical Psychology, Tottori University Graduate School of Medical Sciences, Yonago, 683-8503 Japan; 2 Psychosocial Epidemiology, Graduate School of Nursing, Dokkyo Medical University, Mibu, 321-0293 Japan

**Keywords:** Japanese worker, Negative and positive affectivity, Psychological distress, Work engagement, Work environment

## Abstract

**Background:**

A previous study has shown that Japanese individuals generally exhibit behavior that suppresses the expression of positive emotions, which are strongly affected by affectivity traits. In the present study, to clarify the relationship between affectivity traits and work engagement (WE) or work-related psychosocial factors among Japanese workers, we compared it to the association between psychological distress and these same factors.

**Methods:**

A total of 1,000 full-time Japanese regular workers responded to an online survey that measured demographic variables, negative and positive affectivity, job demands and resources, WE, and psychological distress. A hierarchical multiple regression analysis was conducted separately, which used WE and psychological distress as dependent variables.

**Results:**

The proportion of variance explained by negative and positive affectivity was lower for WE than for psychological distress. However, the proportion of variance defined by job demands and resources was higher for WE than for psychological distress. The proportion of variance explained by all variables for negative and positive affectivity and job demands and resources, and their interactions was approximately equal for WE and psychological distress.

**Conclusion:**

These results emphasize when researchers aim to evaluate the change of psychosocial factors in the workplace, such as improving the workplace environment among Japanese workers, it might be beneficial to measure positive indicators in addition to negative indicators. Furthermore, enriching job resources would be effective in improving WE and alleviating psychological distress.

**Supplementary Information:**

The online version contains supplementary material available at 10.1186/s40359-023-01250-y.

## Background

Work engagement (WE) refers to “a positive, fulfilling, work-related state of mind characterized by vigor, dedication, and absorption” [1] and is beneficial for both individual workers and organizations. Studies have indicated that WE is associated with improved physical and mental health [2, 3]. Furthermore, WE is associated with job performance [2, 4] and business growth [5]. Therefore, several researchers have developed intervention programs and verification methods in the workplace environment to enhance WE [6, 7].

To improve the mental health of employees, industrial and organizational psychologists and practitioners who evaluate either the psychosocial environments in the workplace or the effect of workplace intervention programs ought to ensure the accurate and sensitive documentation of job stressors as well as the mind and body state of employees [8]. Furthermore, the influence of individual factors, such as sociodemographic variables and worker personalities, should be considered to a significant extent. However, it is widely known that individual characteristics are associated with stress responses [9–11]. For example, negative affectivity (NA) of affective dispositions [12, 13] is generally known that a personality trait strongly associated with work environment factors and stress responses [14, 15]. Therefore, statistically controlling NA had been recommended to distinctly determine the relationship between job stressors and stress responses [16, 17]. On the other hand, scholars have cautioned that statistical control removes true variance and distorts the effects of causal variables (e.g., job stressors and stress responses) and is thus undesirable [18]. Thus, the influence of a worker’s individual characteristics is exceedingly and intricately associated with workplace environmental factors or the worker’s stress response.

Similarly, this association might also be the case for the WE. The job demands-resources model includes WE as part of its motivational process [19, 20]. However, in the job demand-resource model, although individual characteristics are included in personal resources, their position remains indefinite [20]. Young et al. [21], conducting a meta-analysis of the association between personality traits and WE, demonstrated that among various personality traits, positive affectivity (PA) was most strongly associated with WE.

Watson and Tellegen [22] found that the various moods nursed by humans can be categorized into two domains, namely, negative and positive affect. Subsequently, Watson et al. [13] discovered that negative and positive affect are pertinent in both state and trait ratings and thus referred to these trait domains as NA and PA. High NA describes the ease of evoking negative emotions such as “scary” or “sluggish,” while high PA refers to the ease of evoking positive emotions such as “energetic” or “lively” [13]. This two-dimensional factor structure is common in Japan, the United States, and Europe [23], and the NA and PA roughly correspond to the dominant personality traits of neuroticism and extraversion in a five-factor model, respectively [24].

A study comparing WE measurements between Japanese and Dutch people ought to consider cultural differences when interpreting WE values as Japanese people tend to suppress positive emotions while self-enhancement for Dutch people can represent lower measurement accuracy [25]. Furthermore, studies have shown that the Japanese are more likely to suppress expressions of positive emotions in contrast to Europeans and Americans due to cultural customs [26–28]. Iwata et al. [29] compared the factor structure of the State-Trait Anxiety Inventory (STAI) between Japanese and Western individuals and found that the affectivity traits of Japanese people largely determined positive emotions. Thus, WE measurements among Japanese individuals exhibit a stronger reflection of their affectivity traits in contrast to Europeans and Americans.

Therefore, the purpose of this study was to clarify the relationship between trait affectivity and WE or work environment factors among Japanese workers. For the above reasons, the WE of Japanese workers would strongly reflect the influence of affectivity. To test this hypothesis, in addition to examining the association between WE and affectivity factors or work environment factors, this study will examine the association between psychological distress and affectivity factors or work environment factors. Previous studies show that there is no cultural difference between Japanese and Europeans or Americans in terms of stress responses comprising negative aspects such as depression and anxiety [26, 28, 30]. Thus, by comparing the results of psychological distress without cultural differences and WE with cultural differences, the extent to which affective traits influence the association between WE and work environmental factors can be clarified.

### Hypothesis 1

The proportion explained by affectivity factors is higher in WE variance than in psychological distress for Japanese workers.

WE seems to be strongly influenced by work environment factors. Studies examining the relationship between workplace environmental factors and WE have been actively conducted in many countries. These studies have consistently implied that measures to enrich job resources such as job control or social support in the workplace are essential for improving WE [31, 32]. Furthermore, previous studies have shown that not only job resources but also job demands are associated with WE [33–35].

The job demands-resources model shows that when job resources are low, WE decreases and stress reactions increase [19, 20]. Furthermore, WE has been found to mediate the relationships between job demands and psychological distress [19, 20]. In other words, WE can be considered to influence psychological distress. Although it is widely known that psychosocial factors in the workplace are related to psychological distress [36–39], the effects of those factors would be stronger on WE than on psychological distress.

### Hypothesis 2

The proportion explained by work environmental factors is higher in WE variance than in psychological distress variance.

We test the above two hypotheses. To our knowledge, no empirical study has been conducted that simultaneously measures and examines in detail the relationships between affectivity traits, work environmental factors, and WE.

## Methods

### Study design and data collection

A cross-sectional survey was conducted among registered marketing research monitors and an internet survey firm (Rakuten Insight, Inc). Thus, the participants provided their data using the internet. The inclusion criteria of the participants were as follows: (a) Japanese and (b) full-time employees of the organization. Self-employed, part-time, and unemployed workers were excluded from this study. The Internet survey company recruited monitor workers until the target number was reached based on the inclusion criteria. The recruited workers were able to access a self-report questionnaire of the present study.

### Participants

To increase the likelihood of obtaining a representative sample of Japanese workers, the population was assigned proportions according to gender, age, and place of residence based on population estimates published by the Statistics Bureau of the Ministry of Internal Affairs and Communications [40]. Furthermore, to minimize gender differences in the analysis, the gender proportions were set to be equal. Data were obtained from 1,000 Japanese workers (i.e., 504 men and 496 women). The mean age of the participants was 45.6 years (standard deviation, 13.0).

### Measures

#### Affectivity traits

The negative and positive affectivity of the participants was measured using the Japanese version of the Positive and Negative Affect Schedule (PANAS) [41]. This version of the PANAS consists of 16 items: eight items for negative affect and eight items for positive affect. Typically, the PANAS requires respondents to rate the frequency of their feelings over four weeks on a six-point scale ranging from 1 (never) to 6 (always). However, this study focused on measuring stable traits of both negative and positive affect. Thus, the PANAS instructions were revised from “How often have you felt these moods in the past month?” to “To what extent do you usually feel these moods?” The items were scored on a six-point scale ranging from 1 (totally disagree) to 6 (totally agree).

#### Work-related psychosocial factors

Job demands and resources were measured using the Brief Job Stress Questionnaire (BJSQ) [42]. “Job demands” comprised three items for both the quantitative and the qualitative workload. The items were scored on a four-point scale ranging from 1 (very much) to 4 (not at all). “Job resources” consisted of nine items, namely, three for job control and six for support from supervisors and co-workers. All job resources items were scored on a four-point scale ranging from 1 (very much) to 4 (not at all). A high score for job demands indicated a high workload while a high score for job resources indicated extensive workplace resources.

#### Work engagement

The WE among the participants was assessed using the Japanese version of the Utrecht Work Engagement Scale (UWES) [43]. The UWES consists of three subscales (i.e., vigor, dedication, and absorption); each comprise three items scored on a seven-point scale ranging from 0 (never) to 6 (always). The overall score for the UWES is the sum of the three subscales.

#### Psychological distress

The Kessler 6 (K6) scale [44, 45] was used to measure psychological distress. K6 requires respondents to describe how frequently they have experienced each statement during the past 30 days. The items were scored on a five-point scale ranging from 0 (none of the time) to 4 (all of the time).

#### Demographic variables

Several variables were analyzed in the questionnaire, namely, age, gender, educational background, marital status, number of children, occupation, duration in the current job, and night shift.

### Statistical analysis

First, the correlation coefficients between each variable and Cronbach’s alpha coefficients were estimated. Thereafter, a hierarchical multiple regression analysis of WE and psychological distress was performed before entering the independent variables in Model 1 in the following order: age, gender, and career in the current job. In Model 2, job demands and resources were used as occupational factors while affective factors were used in Model 3. In Model 4, the two two-way interactions (job demands × PA or NA, and job resources × PA or NA) were inserted to analyze the interactive effects between occupational and affective factors [46]. Finally, the interactions were calculated after centering each variable using its mean to account for multicollinearity issues. The statistical analyses were performed using R, version 4.1.0.

## Results

### Preliminary analyses

Table 1 shows the demographic characteristics of the participants. Approximately 60% of the participants held university or graduate school degrees. Furthermore, it was estimated that 70% of the participants were non-manual workers.

**Table 1:** Characteristics of respondents (*N* = 1000)

	*N*	%
Gender		
Men	504	50.4
Women	496	49.6
Education		
University/graduate school graduate	589	58.9
Vocational school/college graduate	221	22.1
High school graduate	179	17.9
Junior high school graduate	8	0.8
Others	3	0.3
Marital status		
Unmarried	319	31.9
Married	576	57.6
Divorce	93	9.3
Bereavement	12	1.2
Number of child(ren)		
0	467	46.7
1	150	15.0
2	277	27.7
3	97	9.7
4	9	0.9
Occupations		
Managers	181	18.1
Non-manual workers	674	67.4
Manual workers	68	6.8
Others	77	7.7
Night shift		
Yes	139	13.9
No	861	86.1

Table 2 shows the correlation coefficients between the variables with Cronbach’s alpha coefficients. This study found moderate associations between age and career in terms of the current job, NA and psychological distress, PA and WE, as well as job resources and WE. Cronbach’s alpha coefficients were greater than 0.80 for all variables.

**Table 2:** Correlations and reliability estimates for study variables (Cronbach’s alpha) (*N* = 1000)

		Mean	SD	1	2	3	4	5	6	7	8
1	Age	45.6	13.0	ー							
2	Career in the current job(yrs)	13.8	11.5	0.57***	ー						
3	Negative affectivity	23.4	7.7	-0.21***	-0.09**	(0.90)					
4	Positive affectivity	24.7	6.5	0.01	0.02	0.05	(0.87)				
5	Job demands	16.2	3.8	-0.13***	-0.08*	0.26***	0.09**	(0.84)			
6	Job resources	23.4	5.2	-0.03	0.02	-0.29***	0.31***	-0.03	(0.86)		
7	Work engagement	23.0	11.8	0.19***	0.11***	-0.26***	0.51***	0.13***	0.46***	(0.96)	
8	Psychological distress	6.0	5.3	-0.23***	-0.14***	0.64***	-0.15***	0.27***	-0.32***	-0.31***	(0.90)

*SD* Standard deviation

^*^p < 0.05, **p < 0.01, ***p < 0.001

### Hierarchical multiple regression analysis

Tables 3 and 4 show the hierarchical multiple regression analysis results, with WE and psychological distress as the respective dependent variables.

**Table 3:** Hierarchical multiple regression analysis with work engagement as the dependent variables (*N* = 1000)

	Model 1			Model 2			Model 3		Model 4	
	*β*		95%CI	*β*	95%CI		*β*	95%CI	*β*	95%CI
Demographics										
Age	0.19***		0.11―0.26	0.24***	0.17―0.30		0.17***	0.12―0.23	0.17***	0.12―0.23
Gender (1 = Men, 2 = Women)	0.03		-0.03―0.09	0.05	-0.00―0.10		0.07**	0.02―0.11	0.07**	0.02―0.11
Career in the current job (yrs)	0.00		-0.07―0.08	-0.02	-0.08―0.04		-0.01	-0.06―0.05	-0.01	-0.06―0.05
Occupational factors										
Job demands				0.17***	0.12―0.22		0.17***	0.12―0.22	0.17***	0.12―0.22
Job resources				0.47***	0.42―0.52		0.28***	0.23―0.33	0.28***	0.23―0.33
Affective factors										
Positive affectivity							0.42***	0.37―0.47	0.41***	0.36―0.46
Negative affectivity							-0.20***	-0.25―-0.15	-0.20***	-0.25―-0.15
Occupational factors x Affective factors										
Job demands x Positive affectivity									0.02	-0.03―0.06
Job resources x Positive affectivity									-0.01	-0.05―0.03
Job demands x Negative affectivity									-0.03	-0.07―0.01
Job resources x Negative affectivity									-0.02	-0.06―0.03
*ΔR*^*2*^	0.04			0.24***			0.17***		0.00	
*Adjusted R*^*2*^	0.03***			0.28***			0.45***		0.45***	

*β* Standardized regression coefficients, *R*^*2*^ Coefficient of determination, *CI* Confidence interval

^**^p < 0.01; ***p < 0.001

**Table 4:** Hierarchical multiple regression analysis with psychological distress as the dependent variables (*N* = 1000)

	Model 1		Model 2		Model 3		Model 4	
	*β*	95%CI	*β*	95%CI	*β*	95%CI	*β*	95%CI
Demographics								
Age	-0.22***	-0.29―-0.14	-0.20***	-0.27―-0.14	-0.07*	-0.13―-0.01	-0.07*	-0.12―-0.01
Gender (1 = Men, 2 = Women)	-0.02	-0.08―0.04	-0.04	-0.09―0.02	-0.02	-0.06―0.03	-0.01	-0.06―0.03
Career in the current job (yrs)	-0.02	-0.09―0.06	0.00	-0.07―0.06	-0.03	-0.09―0.02	-0.04	-0.09―0.02
Occupational factors								
Job demands			0.23***	0.18―0.29	0.12***	0.07―0.17	0.11***	0.06―0.16
Job resources			-0.32***	-0.37―-0.26	-0.11***	-0.16―-0.06	-0.11***	-0.16―-0.06
Affective factors								
Positive affectivity					-0.15***	-0.20―-0.10	-0.14***	-0.19―-0.09
Negative affectivity					0.57***	0.52―0.62	0.56***	0.51―0.61
Occupational factors x Affective factors								
Job demands x Positive affectivity							-0.03	-0.07―0.01
Job resources x Positive affectivity							0.03	-0.01―0.06
Job demands x Negative affectivity							0.02	-0.02―0.06
Job resources x Negative affectivity							-0.06**	-0.10―-0.02
*ΔR*^*2*^	0.05		0.16***		0.26***		0.01**	
*Adjusted R*^*2*^	0.05***		0.21***		0.47***		0.48***	

*β* Standardized regression coefficients, *R*^*2*^ Coefficient of determination, *CI* Confidence interval

^*^p < 0.05; **p < 0.01; ***p < 0.001

In Model 1, only age was positively associated with WE. Furthermore, including occupational factors in Model 2 resulted in a significant increase in the coefficient of determination (Δ*R*^2^ = 0.24). Moreover, age, job demands, and resources were all positively associated with WE. Model 3 includes affective factors and a significant increase was observed in the determination coefficient (Δ*R*^2^ = 0.17). Thus, the WE variances associated with occupational and affective factors were higher for occupation factors than for affective factors. Furthermore, age, gender, job demands, resources, and PA were positively associated while NA was negatively associated with WE. Regarding the change in the standardized regression coefficient of occupational factors in Models 2 and 3, job resources decreased (*β* = 0.19) despite the absence of change in job demands. In Model 4, the coefficient of determination did not increase significantly and no interactions were associated with WE.

**Fig. 1:**
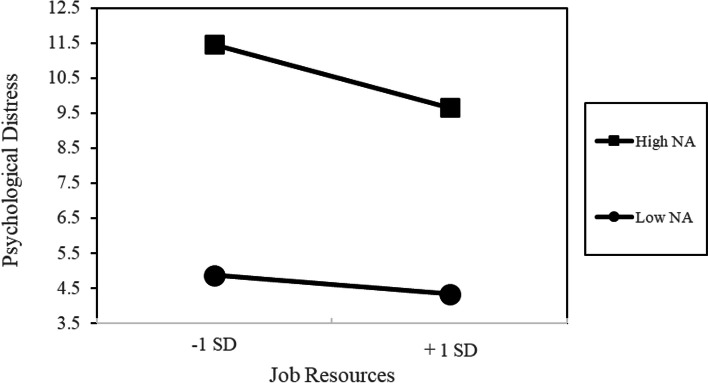
Interaction effect of job resources and negative affectivity on psychological distress

In the context of psychological distress, age was negatively associated with psychological distress in Model 1. The inclusion of occupational factors in Model 2 resulted in a significant increase in the coefficient of determination (Δ*R*^2^ = 0.16). Furthermore, job demands in Model 2 were positively associated while age or job resources were negatively associated. In Model 3, the affective factors were introduced and the coefficient of determination increased significantly (Δ*R*^2^ = 0.26). Thus, the variances in psychological distress explained by occupational and affective factors were more significant for the affective factors in contrast to the occupational factors. Moreover, age, job resources, and PA were negatively associated, while job demands and negative affectivity were positively associated. For the change in the standardized regression coefficient of occupational factors in Models 2 to 3, a decrease was found in job demands and resources (job demands: *β* = 0.11, resources: *β* = 0.21). In Model 4, the coefficient of determination increased significantly (Δ*R*^2^ = 0.01). A simple slope analysis showed that psychological distress did not change with job resources when NA was low (mean － 1 SD). However, when NA was high (mean + 1 SD), job resources significantly reduced psychological distress (see Fig. 1).

Finally, comparing the results in Tables 3 and 4, the proportion explained by affectivity factors was higher in psychological distress than in WE (WE; Δ*R*^2^ = 0.17, Psychological distress; Δ*R*^2^ = 0.26). Conversely, the proportion explained by occupational factors was higher in WE than in psychological distress (WE; Δ*R*^2^ = 0.24, Psychological distress; Δ*R*^2^ = 0.16). Excluding the association of demographic variables, the proportion explained by all variables including occupational and affectivity factors and their interactions in Models 2 to 4 nearly equal in variances of WE and psychological distress (WE; Δ*R*^2^ = 0.41 or adjusted *R*^*2*^ = 0.42, Psychological distress; Δ*R*^2^ = 0.43 or adjusted *R*^*2*^ = 0.43).

## Discussion

The purpose of this study was to clarify the relationship between trait affectivity and WE or work environment factors among Japanese workers. The results indicated that the proportion of variance explained by positive and negative affectivities was lower for WE than for psychological distress, and thus hypothesis 1 was not supported. Second, the proportion of variance explained by job demands and resources was higher for WE than for psychological distress, thus supporting hypothesis 2.

In the present study, affective factors accounted for nearly 20% of the variance in the WE. Furthermore, the percentage of variances in WE associated with affective traits was approximately 30% (∆*R*^2^ = 0.324) despite changing the input order of the occupational and affective factors in Models 2 and 3. The results suggest that the influence of affective factors on WE is small when compared to the meta-analysis [21], which found that negative and positive affectivities explained nearly 40% of the variance in WE. Contrastingly, even when comparing the effects of psychological distress in the present study, the influence of affective factors on WE was small. Thus, the result differed from Iwata et al. [29]. The current results suggest that it could be more effective to focus on workplace environmental factors such as job demands or resources, rather than on individual factors such as affectivity traits for WE among Japanese workers.

High extraversion and low neuroticism tendencies are important in enhancing WE [47, 48]. Young et al. [21] found that PA accounted for 90.6% of the total proportion of both NA and PA, explaining the variance in WE (i.e., 9.4% for NA and 90.6% for PA). We entered the affective factors one by one and re-calculated the NA and PA ratio for ∆*R*^*2*^ in Model 3 of Table 3. The results showed that the PA and the NA were 80.8% and 19.2%, respectively. In other words, the proportion of NA was higher than the findings reported by Young et al. [21]. Several international studies comparing the five-factor model have reported high neuroticism as one of the typical personality traits among Japanese individuals [49, 50]. Particularly, Japanese people have stronger NA than individuals from other countries. Therefore, the affective factor that characterizes the high WE of Japanese workers is a high level of PA and is expressed as low NA as an essential characteristic.

Job demands and resources explained the higher percentage of the variance observed in WE as opposed to psychological distress. The change in standardized regression coefficients on job demands and resources in Model 3 (Tables 3 and 4, respectively) with affectivity factors as an input was smaller for WE than for psychological distress, and the proportion explained by affectivity traits for the variance of WE was smaller than for the variance of psychological distress. These results indicate that the association between WE and job demands or resources is less influenced by affectivity traits than the association between psychological distress and that for Japanese workers. Therefore, when researchers aim to accurately and sensitively evaluate the change of psychosocial factors in the workplace, such as improving the workplace environment among Japanese workers, it might be beneficial to measure positive indicators (e.g., WE) in addition to negative indicators (e.g., psychological stress responses).　Because, positive indicators, such as WE, are less influenced by individual factors such as affective traits, and those indicators strongly reflect the influence of work environment factors.

Studies have consistently implied that measures to enrich job resources are essential for improving WE [31, 32]. Model 3 (Tables 3 and 4) shows that job resources significantly impacted all dependent variables and were more influential on WE than job demands when the demographic variables and affective factors were controlled. Similarly, job demands and resources have an equal influence on psychological distress. These findings indicate that enriching job resources can reduce psychological distress and improve WE regardless of the affective traits of the worker. Furthermore, the results of the interaction between NA and job resources on psychological distress indicate that the psychological distress of workers with higher NA is mitigated by many job resources in the workplace although they are likely to experience psychological distress [51, 52]. Therefore, enriching job resources would be effective in improving WE and alleviating psychological distress.

### Limitations

This study has several limitations. First, the causal relationships between variables could not be addressed as this was a cross-sectional study. Therefore, in the future, we need to conduct a longitudinal study to verify whether the findings of this study are robustly replicated. Second, the participants in this study were all registered monitors chosen by the same internet survey company; thus, selection bias might have affected the results. Third, the variable of job demands and resources used in this study were ones included in the job demands-control-support model [53]. However, given the existence of several other variables for job demands and resources [20], it is necessary that verification be based on other variables not used in this study. Fourth, although we conducted the statistical analysis of the interaction between work environment factors and affectivity traits, we have not been able to conduct a detailed examination on why there is an interaction between negative affectivity and job resources in psychological distress and no interaction in other combinations. Finally, this survey was conducted via the Internet in November 2020, during the coronavirus 2019 (COVID-19) pandemic. Furthermore, some of the companies where the study participants worked might have been operating remotely to prevent the spread of infection. Therefore, the changes in work patterns and daily lifestyles might have influenced the findings of this study.

## Conclusion

This study indicated that for Japanese workers, the association between WE and job demands or resources was less influenced by affectivity traits than the association between psychological distress and them. Thus, these results emphasize when researchers aim to evaluate the change of psychosocial factors in the workplace, such as improving the workplace environment among Japanese workers, it might be beneficial to measure positive indicators in addition to negative indicators.　Furthermore, enriching job resources would be effective in improving WE and alleviating psychological distress.

### Supplementary Information

**Additional file 1: Appendix 1.** The factor structure of PANAS in Japanese workers (*N* = 1000).

## Data Availability

The datasets used and/or analysed during the current study are available from the corresponding author on reasonable request.

## Abbreviations

BJSQ: Brief job stress questionnaire
COVID-19: Coronavirus 2019
K6: Kessler 6
NA: Negative affectivity
PA: Positive affectivity
PANAS: Positive and negative affect schedule
STAI: State-trait anxiety inventory
UWES: Utrecht work engagement scale
WE: Work engagement
